# Increased Prevalence of Psychiatric Disorders in Children with RASopathies: Comparing NF1, Noonan Syndrome Spectrum Disorder, and the General Population

**DOI:** 10.3390/genes16070843

**Published:** 2025-07-19

**Authors:** Yaffa Serur, Odeya Russo, Chloe Alexa McGhee, Tamar Green

**Affiliations:** Division of Interdisciplinary Brain Sciences, Department of Psychiatry and Behavioral Science, School of Medicine, Stanford University, Stanford, CA 94305, USA; yaffaserur@gmail.com (Y.S.); odeya@stanford.edu (O.R.); camcghee@stanford.edu (C.A.M.)

**Keywords:** RASopathies, Neurofibromatosis type 1, Noonan syndrome spectrum disorder, psychiatric disorders

## Abstract

**Background/Objectives**: Neurofibromatosis type 1 (NF1) and Noonan syndrome spectrum disorders (NSSD) are the most common RASopathies, resulting from germline mutations that affect the RAS-MAPK signaling pathway. Both are associated with increased risk for neurodevelopmental and psychiatric conditions, yet few studies have used structured diagnostic interviews to compare their psychiatric comorbidities. **Methods:** We conducted clinician-administered DSM-5 diagnostic assessments (KSADS) in 123 children with RASopathies (NF1 = 29, NSSD = 94; ages 5–15). Diagnosis prevalence was compared within each group and to population-based estimates. **Results:** Psychiatric diagnoses were highly prevalent, at 79.3% in NF1 and 76.6% in NSSD, with ADHD (NF1 = 72.4%, NSSD = 51.1%) and anxiety disorders (NF1 = 37.9% and NSSD = 43.6%) being the most common, rates substantially higher than those reported in general population estimates. Behavioral and sleep disorders were identified in approximately 25% of both groups. Notably, social anxiety disorder was identified in 14.9% of NSSD but not in NF1. Full-scale IQ did not significantly differ by diagnosis status. Specific anxiety disorders, elimination disorders, obsessive–compulsive disorder, and post-traumatic stress disorder were characterized, expanding the known psychiatric phenotype of RASopathies. **Conclusions:** Children with NF1 and NSSD demonstrate similarly high rates of ADHD, anxiety, and behavioral disorders compared to the general population; in addition, we report sleep disorders in NSSD and characterize psychiatric disorders not previously described in RASopathies. The shared psychiatric profiles may reflect the common effect of RAS-MAPK pathway dysregulation on psychiatric outcomes. These findings highlight the need for early, syndrome-informed mental health screening and intervention in the clinical care of individuals with RASopathies.

## 1. Introduction

RASopathies are a group of developmental disorders caused by germline mutations in genes encoding components of the RAS-mitogen-activated protein kinase (RAS-MAPK) signaling pathway, a molecular cascade critical for regulating cell proliferation, differentiation, and survival [1]. Hyperactivation of this pathway leads to a spectrum of syndromic conditions that share overlapping somatic features, such as short stature, facial dysmorphisms, cardiovascular anomalies, and increased cancer risk, yet exhibit distinct genetic and phenotypic profiles [2,3]. Among the RASopathies, Neurofibromatosis type 1 (NF1) and Noonan syndrome (NS) are the most prevalent. NF1 affects approximately 1 in 3000 individuals and results from mutations or deletions in the *NF1* gene located on chromosome 17, which encodes neurofibromin, a tumor suppressor that negatively regulates RAS-MAPK signaling [4]. NS is estimated to occur in approximately 1 in 1000 to 2500 live births and is most frequently associated with mutations in *PTPN11*, a gene encoding a protein tyrosine phosphatase that regulates RAS signaling. Additional NS spectrum disorder (NSSD)-related genes include *SOS1*, *KRAS*, *RAF1*, and others, which contribute to phenotypic variability [5]. NF1 is characterized by the presence of café-au-lait macules, neurofibromas, Lisch nodules, and optic pathway gliomas and has a well-established risk for both benign and malignant tumors [4]. NSSD is characterized by distinctive facial features, short stature, chest deformity, congenital heart disease, and other comorbidities [6].

In addition to their somatic manifestations, both NF1 and NSSD are increasingly recognized as neurodevelopmental syndromes associated with a high prevalence of cognitive, behavioral, and psychiatric challenges. Children with NF1 exhibit elevated rates of attention deficit/hyperactivity disorder (ADHD), anxiety disorders, learning disabilities, and autism spectrum disorder (ASD) symptoms, with some studies estimating that up to 50–70% of affected individuals meet the criteria for one or more psychiatric conditions [7,8]. NSSD is similarly associated with attention, social, and emotional difficulties, including high rates of ADHD, anxiety, and ASD features. However, prevalence estimates vary widely depending on assessment method and sample size. While these findings point to overlapping psychiatric vulnerabilities across NS and NF1, existing research is limited by a narrow diagnostic focus, inconsistent assessment tools, and small sample sizes. A few studies have employed DSM-5-based diagnostic approaches in children with either NF1 or NS [9,10]; however, these efforts have typically focused on specific disorders (e.g., neurodevelopmental disorders) and may not fully reflect the broader range of less-reported psychiatric comorbidities. Significantly, no study to date has directly compared psychiatric disorder profiles across both syndromes and in relation to the general population using standardized clinical interviews. A direct, comprehensive comparison can enhance our understanding of the psychiatric features most prevalent within each syndrome, those shared between NF1 and NSSD and potentially generalizable across RASopathies, and those that may be syndrome-specific. These insights can help improve early identification, guide syndrome-informed screening practices, and support the development of more personalized treatment strategies across RASopathies. This study aims to fill this gap by conducting a comprehensive, cross-syndrome assessment of psychiatric disorder prevalence in children with NS and NF1, compared both to each other and to the general population, using gold-standard diagnostic methods.

The goal of this study is to systematically assess the prevalence of DSM-5 psychiatric disorders in children with NF1 and NSSD using structured clinical diagnostic interviews. By applying a uniform assessment framework across both groups, we aim to achieve the following: 1. Determine the overall psychiatric comorbidity within each syndrome (NF1 and NSSD). This will help quantify the burden of psychiatric disorders in each condition to inform clinical care and resource allocation. 2. Characterize and compare the specific diagnostic profiles most commonly associated with NF1 and NSSD. This comparison will identify shared and syndrome-specific vulnerabilities and guide targeted interventions. 3. Evaluate how the prevalence of psychiatric disorders in these groups compares to estimates from the general pediatric population. This will contextualize psychiatric risk in NF1 and NSSD and highlight areas of elevated concern relative to the age-matched general population.

## 2. Materials and Methods

### 2.1. Participants

With approval from the Institutional Review Board of the Stanford University School of Medicine, we conducted a prospective study of children aged 5 to 15 years with NF1 (N = 29, mean age = 8.97, females = 14) and NSSD (N = 94, mean age = 9.83, females = 48). The NF1 and NS groups were matched on age, sex, and Full-Scale IQ (FSIQ) to allow for more accurate cross-syndrome comparisons. Upon participation, 26 NF1 participants provided genetic reports verifying germline pathogenic variants in NF1, one NF1 participant presented with an NF1 microdeletion, and two NF1 participants presented with a clinical diagnosis. Similarly, children with NS presented clinical genetic information, confirming the status of pathogenic variants including *PTPN11* (N = 66), *SOS1* (N= 8), and other NS mutations (N= 20) such as *LZTR1*, *NRAS, SOS2, RAF1*, *RIT1*, and *KRAS*, in addition to *SHOC2,* associated with Noonan-like syndrome with loose anagen hair (NS/LAH) and *PTPN11*-gain-of-function mutations associated with NSML (*PTPN11*/NSML). The demographic characteristics of the study sample, including medication status, are presented in Table 1. All participants were included in the analyses, regardless of whether they were receiving psychiatric medication at the time of assessment. For comparison with the general population, we utilized mental health data from the 2022–2023 National Survey of Children’s Health (NSCH), which was administered to children aged 3–17 years. Additional details regarding participants, recruitment, written consent, exclusion criteria, and general population data can be found in the Supplementary Materials.

### 2.2. Cognition

Cognition was assessed using age-appropriate versions of the Wechsler Abbreviated Scale of Intelligence (WASI, [11]). Of the 123 participants who completed the WASI, 95 completed the standard four-subtest version, and 27 completed a two-subtest version administered remotely, as certain subtests requiring physical materials or in-person interaction could not be completed virtually [12]. One participant had a missing FSIQ measure.

### 2.3. Psychiatric Disorders

To assess psychiatric disorders in children with NF1 and NS, we used the Kiddie Schedule for Affective Disorders and Schizophrenia for DSM-5 (KSADS) [13]. The KSADS is a semi-structured interview widely used in clinical and research settings to assess current and past episodes of psychopathology in children and adolescents, based on DSM criteria [13]. Although the KSADS is designed for children aged 6 and older, we administered it to participants aged 5 to 17 years in our study, consistent with prior research protocols. The instrument was updated in 2013 to align with DSM-5 diagnostic criteria and is now available in computerized formats [14], including a clinician-administered version, a self-administered parent version (parent-report on youth), and a self-administered youth version (self-report). In this study, each participant completed both the clinician-administered KSADS-5 and the parent-report version. The clinician interviews were conducted by doctoral-level psychology trainees under the supervision of a licensed clinical psychologist. Diagnostic results from both versions were reviewed in a consensus meeting with a child and adolescent psychiatrist (Y.S.) to establish a final diagnosis. Psychiatric disorders included in the assessments were mood disorders, anxiety disorders (including generalized anxiety disorder (GAD), separation anxiety, social anxiety, and specific phobia), attention deficit hyperactivity disorder (ADHD), obsessive–compulsive disorder (OCD), elimination disorders, sleep disorders, behavioral disorders (oppositional defiance disorders and conduct disorders), tic disorders, and post-traumatic stress disorder (PTSD).

### 2.4. Statistical Analysis

All statistical analyses were performed in R v4.4.1. (R Core Team, 2024). We examined demographic characteristics, including age, sex, and FSIQ, by comparing the NF1 and NSSD groups. FSIQ was compared between groups using a two-sample t-test, and sex distribution was analyzed using a chi-square test. As age was non-normally distributed, group comparisons for age were conducted using the Mann–Whitney U test.

#### Psychiatric Diagnoses Analysis

Psychiatric conditions were assessed using the KSADS, which yields categorical (yes/no) diagnostic outcomes for each disorder. For each diagnostic category, we calculated the prevalence within each genetic group (NF1 and NSSD) by dividing the number of children meeting diagnostic criteria by the total number of children in that group. FSIQ differences between children with and without each psychiatric diagnosis were assessed using two-sample t-tests. Before conducting these tests, we evaluated the normality of FSIQ distributions using the Shapiro–Wilk test, which is a prerequisite for parametric analysis. However, this test is not reliable for groups with fewer than three observations. Therefore, diagnoses with fewer than three cases were excluded from analysis, as normality could not be assessed and t-tests could not be validly applied. Differences in the prevalence of psychiatric disorders between the NF1 and NSSD groups were tested using Fisher’s exact tests. Given the multiple comparisons across psychiatric conditions, we applied the Bonferroni correction to control for false positives and reduce the likelihood of Type I errors.

To assess whether the prevalence of psychiatric conditions in the NF1 and NSSD groups differed from general population estimates in U.S. children, we conducted binomial tests. Specifically, we compared prevalence rates from our study data, including any psychiatric disorder as well as five key diagnostic categories (ADHD, mood disorders, anxiety disorders, behavioral disorders, and sleep disorders), with prevalence estimates reported in nationally representative U.S. samples [15]. Bonferroni correction was applied to adjust for multiple comparisons and reduce the risk of Type I error.

## 3. Results

### 3.1. Rates of Psychiatric Disorders in NF1

**Figure 1:**
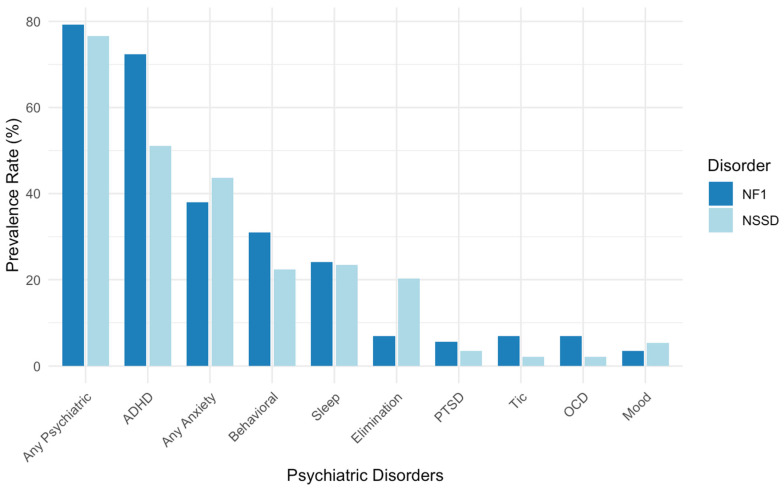
Prevalence of psychiatric disorders in children with NF1 and NSSD. Abbreviations: ADHD, attention deficit hyperactivity disorder; NF1, Neurofibromatosis type 1; NSSD, Noonan syndrome spectrum disorder; OCD, obsessive–compulsive disorder; PTSD, post-traumatic stress disorder.

Among the 29 children with NF1, 79.3% met the DSM-5 diagnostic criteria for at least one psychiatric disorder. The mean number of diagnoses per child was 2.03 (SD = 1.61). The most common diagnoses in this group were ADHD (72.4%) and anxiety disorders (37.9%). Additional diagnoses included behavioral disorders (31.0%), sleep disorders (24.1%), elimination disorders (6.9%), OCD (6.9%), tic disorders (6.9%), mood disorders (3.4%), and PTSD (3.4%). See Figure 1 and Table S1 in the Supplementary Materials for complete diagnostic distributions.

**Figure 2:**
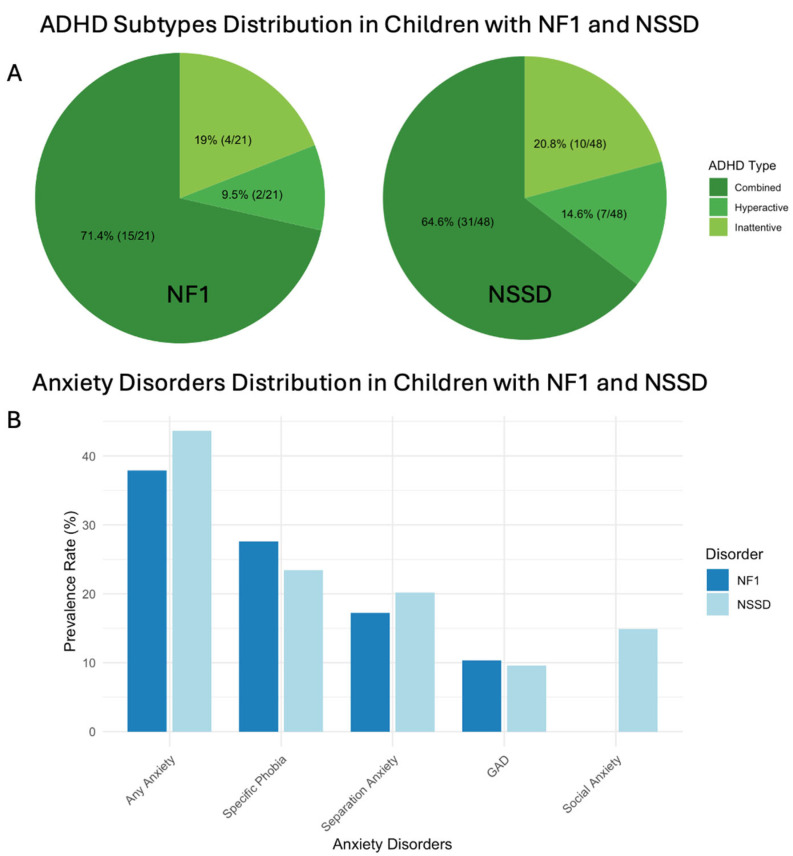
ADHD subtype and anxiety disorder distribution among children with NF1 and NSSD. (**A**) ADHD subtypes among children with NF1 and NS. (**B**) Anxiety disorders distribution among children with NF1 and NSSD. Abbreviations: ADHD, attention deficit hyperactivity disorder; GAD, generalized anxiety disorder; NF1, Neurofibromatosis type 1; NSSD, Noonan syndrome spectrum disorder.

When examining NF1 children diagnosed with ADHD, the combined presentation was most common (71.4%), followed by inattentive (19.0%) and hyperactive/impulsive (9.5%) Across the NF1 group, the most prevalent anxiety disorder was specific phobia (27.6%), followed by separation anxiety (17.2%) and GAD (10.3%). (Table S1, Figure 2). The most commonly reported specific phobias in children with NF1 were fear of darkness, spiders, and injections.

### 3.2. Rates of Psychiatric Disorders in NSSD

Among the 94 children with NSSD, 76.6% met the DSM-5 diagnostic criteria for at least one psychiatric disorder. The average number of diagnoses was 1.95 (SD = 1.78). ADHD was diagnosed in 51.1% of the sample, followed by anxiety disorders (43.6%), sleep disorders (23.4%), behavioral disorders (22.3%), and elimination disorders (20.2%). Other diagnoses included mood disorders (5.3%), PTSD (3.2%), OCD (2.1%), and tic disorders (1.1%). See Figure 1 and Table S1 in the Supplementary Data for diagnostic frequencies.

When examining NSSD children diagnosed with ADHD, the combined presentation was most prevalent (64.6%), followed by inattentive (20.8%) and hyperactive/impulsive (14.6%) (Figure 2). Across the NSSD cohort, the most prevalent anxiety disorder was specific phobia (23.4%), followed by separation anxiety (20.2%), social anxiety (14.9%), and GAD (9.6%) (Table 1 and Table S1, Figure 2). The most common specific phobias reported in children with NSSD were fear of darkness, injections, spiders, animals, and height.

### 3.3. Influence of Cognition on the Prevalence of Psychiatric Disorders

There were no significant differences in FSIQ between participants with or without psychiatric diagnoses, including ADHD, GAD, separation anxiety, social anxiety, behavioral disorders, sleep and elimination disorders, PTSD, tic disorders, OCD, or mood disorders, in either the NF1 or NSSD groups. In the NF1 group, children with higher FSIQ scores showed increased rates of overall anxiety diagnoses (Yes: N = 11, mean FSIQ = 104.82; No: N = 18, mean FSIQ = 95.11; *p* = 0.023, d = 0.85) and specific phobia diagnoses (Yes: N = 8, mean FSIQ = 107.63; No: N = 21, mean FSIQ = 95.43; *p* = 0.007, d = 1.103). These associations approached statistical significance after correction for multiple comparisons (any anxiety: *p* = 0.182; specific phobia: *p* = 0.055). See Table S2 in the Supplementary Materials.

### 3.4. Comparison Between NF1 and NSSD Psychiatric Diagnoses

The overall prevalence of psychiatric diagnoses was similar between the NF1 and NSSD groups. Moreover, differences emerged in the rates of social anxiety disorder, which was not observed in the NF1 group and was present in 14.9% of children with NS (*p* = 0.039). However, these differences did not reach statistical significance after correcting for multiple comparisons (*p* = 0.544). While differences in ADHD did not reach statistical significance (*p* = 0.054), the NF1 group showed a high prevalence of ADHD (72.4%) compared to the NS group (51.1%). See Figure 1 and Table 2 for comparisons across diagnostic categories.

### 3.5. Comparing Psychiatric Prevalence in NF1 and NSSD Relative to the General Children’s Population

**Figure 3:**
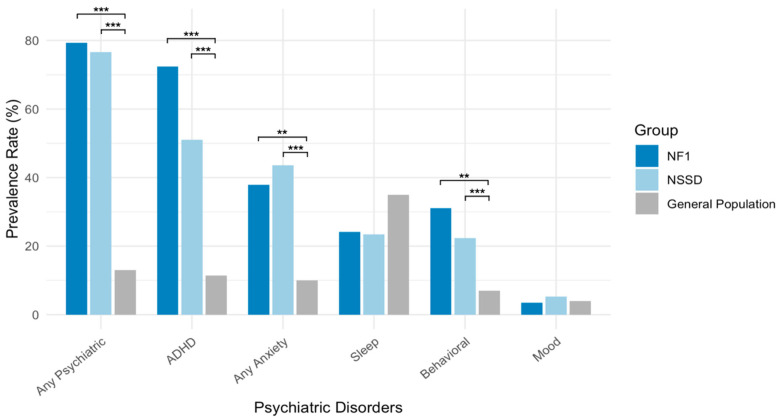
Prevalence of psychiatric disorders in NF1 and NSSD relative to the general children’s population. ** *p* <0.01. *** *p* <0.001. General population data were obtained from the report of the National Survey of Children’s Health (NSCH) [15,16]. Abbreviations: ADHD, attention deficit/hyperactivity disorder; NF1, Neurofibromatosis type 1; NSSD, Noonan syndrome spectrum disorder.

Children with both NF1 and NSSD showed significantly higher rates of psychiatric disorders compared to general population estimates. In both groups (NF1 and NSSD), the prevalence of any psychiatric disorder, ADHD, anxiety disorders, and behavioral disorders was significantly higher than the general population after correcting for multiple comparisons (*p* < 0.01). In contrast, the rates of mood disorders and sleep problems did not significantly differ from general population estimates (Table 2, Figure 3).

## 4. Discussion

This study provides the first comprehensive evaluation of DSM-5 psychiatric disorders in children with NF1 and NSSD, using gold-standard clinical interviews to assess a wide range of psychiatric conditions. By applying a uniform diagnostic approach across both syndromes, we aimed to clarify the psychiatric burden and identify common and syndrome-specific patterns in two of the most common RASopathies.

Our study contributes to the growing literature on psychiatric comorbidities in RASopathies by providing a detailed characterization of diagnostic patterns in children with NF1 and NSSD. We highlight three key findings. First, psychiatric comorbidities were highly prevalent in both groups, with over 75% of children meeting the criteria for at least one DSM-5 diagnosis. Second, ADHD and anxiety disorders emerged as the most common conditions, followed by behavioral and sleep disorders, which affected approximately 25% of the sample. Third, while overall rates of psychiatric diagnoses were comparable between NF1 and NSSD, we observed syndrome-specific patterns; notably, social anxiety disorder was present in 14.9% of children with NSSD but was absent in NF1. However, these group differences did not remain significant after correction for multiple comparisons. Importantly, when compared to population-based estimates, both NF1 and NSSD groups showed markedly elevated rates of ADHD, anxiety disorders, behavioral disorders, and overall psychiatric diagnoses, emphasizing the need for increased clinical awareness and early intervention in these populations.

### 4.1. ADHD

Our findings are consistent with prior studies reporting elevated rates of ADHD in both NF1 and NS [9,17,18]. In NF1, ADHD is well-documented [17], with prevalence estimates ranging from 38% to 50%, often accompanied by executive functioning and attention regulation difficulties [19,20,21,22]. Notably, the prevalence observed in our NF1 sample (72.4%) exceeds those previously reported. Similarly, in NS, previous studies have reported a wide range of ADHD prevalence, from 22% to 60% [9,23], with our study identifying a rate of 51.1%. A portion of the NSSD data has been previously reported in our earlier publication [9], which focused on neurodevelopmental symptomatology in NS. The current study builds on those findings, reinforcing and extending the evidence for elevated ADHD risk in NSSD and situates these findings within a more comprehensive, cross-syndrome context.

Our study extends prior work by characterizing ADHD subtypes in both NF1 and NSSD within a relatively large, well-characterized sample of children in a young and narrow age range (5–15 years old). The combined subtype was most common (NF1 = 71.4%, NSSD = 64.6%), followed by the inattentive subtype (NF1 = 19.0%, NSSD = 20.8%). This contrasts with our earlier NS study, where the inattentive type predominated [9]. The difference may reflect the increased sample size, which allowed for more representative ADHD subtypes. In NF1, meta-analytic findings show that inattention has the strongest effect size, but combined symptoms are more consistently reported, possibly due to ascertainment bias, where more overt symptoms are more likely to lead to diagnosis [17]. In the general population, the inattentive ADHD subtype is typically the most prevalent [24]. The higher prevalence of the combined subtype in our sample may therefore suggest that RASopathies could be associated with an increased burden of hyperactivity symptoms, which may be underrecognized in previous studies. Notably, our recent study found that children with RASopathies exhibit significantly higher levels of irritability compared to controls [25]. Since irritability can manifest behaviorally as hyperactivity or impulsivity in the context of ADHD, this may in part explain the higher rates of the combined presentation observed in our sample. Additionally, hyperactivity in RASopathies may reflect underlying disruptions in brain circuits modulated by the RAS-MAPK pathway, particularly those involved in inhibitory control and executive function, further contributing to difficulties with motor regulation and behavioral inhibition [18,26]. Further research is needed to clarify the neurobiological and behavioral mechanisms underlying ADHD subtypes in RASopathies and to determine whether hyperactivity-related symptoms represent a distinct or amplified phenotype in this population.

Our findings suggest that, when assessed with gold-standard diagnostic tools, in a younger sample and with broader genetic phenotype inclusion, the combined ADHD presentation emerges as the most frequent and consistently observable clinical manifestation in both NF1 and NSSD. Future longitudinal studies are needed to confirm the stability of these findings.

The consistently high prevalence of ADHD across both NF1 and NS raises important questions about its role in the neurobehavioral phenotype of RASopathies. ADHD may represent a core symptom linked to disruptions in key neurodevelopmental pathways affected by RAS-MAPK signaling dysfunction [17,23]. This idea is in addition supported by a growing body of evidence showing elevated ADHD symptoms in other genetic syndromes, such as 22q11.2 deletion syndrome, Turner syndrome, and fragile X syndrome, indicating that attentional dysregulation may be a shared downstream effect of specific genetic mechanisms [27,28,29].

Given the association of ADHD in NF1 and NS with impaired social and academic functioning [17,18,30,31], early recognition and intervention are critical. Longitudinal studies are needed to determine whether ADHD symptoms persist across development, and whether they predict later functional outcomes or interact with other psychiatric comorbidities. Future research integrating structured diagnostic interviews, dimensional symptom measures, and genetic data may help disentangle the developmental and etiological pathways contributing to ADHD in RASopathies.

### 4.2. Anxiety Disorders

Anxiety symptoms have been reported in both NF1 and NSSD, though typically at lower rates and less often categorized according to specific DSM-5 diagnoses. Previous studies have estimated the prevalence of anxiety in NF1 at approximately 16% [32] and at around 13% in NS [10,33]. In contrast, our findings reveal substantially higher rates, 47.9% in NF1 and 43.6% in NSSD. Beyond overall prevalence, our study adds novel detail by characterizing the distribution of specific anxiety disorders. In both groups, specific phobia was the most common, followed by separation anxiety. Notably, the third most common diagnosis differed: generalized anxiety disorder (GAD) in the NF1 group and social anxiety disorder in the NSSD group. Social anxiety disorder was absent among children with NF1 yet was present in 14.9% of those with NSSD. While social difficulties are well-documented in both syndromes [30], to our knowledge, no previous studies have reported on social anxiety disorder specifically. This difference may reflect distinct underlying mechanisms of social impairment in NF1 and NSSD and suggests that targeted interventions for social anxiety may be particularly relevant in NSSD. Alternatively, the absence of social anxiety in the NF1 group may be due to the smaller sample size. Future studies are warranted to further investigate the nature and developmental course of anxiety and specifically social anxiety in RASopathies.

### 4.3. Sleep Disorders

Sleep disorders were commonly observed in both groups, affecting approximately 25% of children with NF1 and NSSD. In NF1, sleep problems are well-documented, with studies reporting difficulties initiating and maintaining sleep, as well as poor sleep quality confirmed by polysomnographic findings [34,35]. In contrast, sleep disturbances in NS have been noted only in a few small case–control studies [36,37] and have not been systematically evaluated. To our knowledge, this is the first study to document DSM-5-defined sleep disorders in a clinically characterized sample of children with NSSD. Although the prevalence of sleep disturbances in our RASopathy sample appeared elevated, it was not significantly different from estimates reported in the general pediatric population. This discrepancy may arise from differences in how sleep problems were assessed: our study relied on a structured clinical interview, whereas general population estimates [16] were based on less rigorous criteria and parent-reported concerns, which may have led to inflated prevalence rates. Given the critical role of sleep in neurodevelopment and emotional regulation, our findings underscore the importance of further research to clarify the prevalence, types, and consequences of sleep disturbances in NS using consistent, clinically validated tools. Moreover, the known association between sleep problems and psychiatric comorbidities [38,39] warrants deeper investigation in both NF1 and NSSD. Routine clinical assessment of sleep disturbances should be considered standard practice in the care of children with RASopathies, particularly given their potential to exacerbate cognitive and psychiatric symptoms [38,40].

### 4.4. Behavioral Disorders, Elimination Disorders, OCD, Tic Disorders, and PTSD

In addition to replicating commonly reported psychiatric disorders in RASopathies, such as ADHD and anxiety, this study identified several psychiatric diagnoses that have been largely absent from the existing NF1 and NSSD literature. These include behavioral disorders, elimination disorders, OCD, tic disorders, and PTSD. While these conditions were less frequent than ADHD or anxiety, their presence highlights the broader spectrum of psychiatric comorbidity in RASopathies and suggests the value of comprehensive, disorder-specific assessment protocols. Recognizing these conditions early is essential, as they may require specialized interventions that differ from those used to treat ADHD or anxiety.

The presence of multiple psychiatric diagnoses in most children across both groups highlights the substantial and cumulative impact of psychiatric comorbidity in RASopathies. These findings underscore the need for integrated neurodevelopmental and mental health care, particularly as behavioral symptoms may complicate the management of the underlying medical condition. It is important to note that our NF1 and NS samples primarily consisted of younger children (mean = 9.62, age range = 5–15 years old), which may have influenced the observed diagnostic profile, particularly the lower rates of conditions that more commonly emerge during adolescence, such as mood disorders. Future studies, including those involving older children and adolescents with NF1 and NS, are needed.

### 4.5. Limitations

Several limitations should be noted. First, the sample size for NF1 was smaller than the NSSD group, which may have limited the statistical power to detect specific disorders and additional group differences. Additionally, estimates derived from the smaller NF1 sample may be more variable or inflated and less representative of the broader NF1 population. Second, although the NSSD sample included a range of pathogenic mutations (e.g., *PTPN11, SOS1, RAF1*), the small sample sizes of the individual subgroups limited the ability to meaningfully examine genotype–phenotype associations or differences in psychiatric outcomes across specific genetic variants. Future studies with larger samples are needed to explore those differences. Third, the cross-sectional design does not capture the developmental course or stability of psychiatric diagnoses over time. In addition, psychiatric diagnoses in this study were established through a consensus approach that integrated information from both the parent and child KSADS interviews when available. While this method reflects clinical practice and was necessary given the young age and neurodevelopmental challenges of many participants, we did not retain separate diagnostic data from each informant to allow for direct comparison. Future studies, particularly those including older children and adolescents, could be enriched by analyzing differences between informant sources to better understand patterns of agreement and the unique contributions of each perspective. Finally, this study focused exclusively on NF1 and NSSD, the two most common RASopathies, and does not address psychiatric outcomes in other syndromes within the RAS-MAPK pathway, such as Costello syndrome or cardiofaciocutaneous (CFC) syndrome. Future studies that include a broader range of RASopathies will be important for understanding shared versus distinct psychiatric phenotypes across this group of conditions.

## 5. Conclusions

In conclusion, children with NF1 and NSSD show a high burden of psychiatric comorbidities compared to the general population, particularly ADHD, anxiety disorders, and behavioral disorders. Our findings underscore the importance of comprehensive psychiatric assessment in these populations, with elevated rates likely reflecting the use of structured, clinician-administered diagnostic interviews. While overall patterns of comorbidity were similar across syndromes, exploratory findings, such as the presence of social anxiety disorder in NS and the identification of sleep disorders in both groups, highlight areas for future investigation. These results support the integration of routine psychiatric and sleep screening into clinical care for children with RASopathies and emphasize the need for continued research to inform developmentally and genetically tailored approaches to intervention.

## Figures and Tables

**Table 1:** Study sample demographic characteristics.

	NF1	NSSD	Overall	*p* Values (Chi-Square or *t*-Test or Mann–Whitney U Test)
N	29	94	123	NF1: NSSD
Age (Mean (SD))	8.97 (2.27)	9.83 (2.99)	9.62 (2.85)	0.278
Sex				
Female (%)	14 (48.3%)	48 (51.1%)	62 (50.4%)	0.960
Male (%)	15 (51.7%)	46 (48.9%)	61 (49.6%)	
FSIQ (Mean (SD)) *	98.8 (12.2)	97.1 (13.5)	97.5 (13.2%)	0.535
Gene Variants (%)				
*NF1*	26 (89.65%)			
*NF1*-Other	3 (10.34%)			
*PTPN11*		66 (70.2%)		
*SOS1*		8 (6.5%)		
NSSD-Other		20 (21.27%)		
Medication status (%)				
Any psychoactive medication	12 (41.4%)	18 (19.1%)	30 (24.4%)	
Stimulants	12 (41.4%)	10 (10.6%)	22 (17.9%)	
SSRI	2 (6.9%)	2 (2.1%)	4 (3.3%)	
Atomoxetine	0 (0%)	3 (3.2%)	3 (2.4%)	
Melatonin	0 (0%)	3 (3.2%)	3 (2.4%)	
Clonidine	1 (3.4%)	1 (1.1%)	2 (1.6%)	
Benzodiazepines	0 (0%)	1 (1.1%)	1 (0.8%)	
Buspirone	0 (0%)	1 (1.1%)	1 (0.8%)	
Gabapentin	1 (3.4%)	0 (0%)	1 (0.8%)	
Guanfacine	0 (0%)	1 (1.1%)	1 (0.8%)	
Remeron	0 (0%)	1 (1.1%)	1 (0.8%)	

* One participant (NSSD, 14-year-old, female) had a missing FSIQ value. Abbreviations: NF1, Neurofibromatosis type 1, NF1-Other, individuals who met a clinical diagnosis of NF1 or had an NF1 microdeletion; NSSD, Noonan syndrome; NSSD-Other, all other NSSD pathogenic variants in the study besides *PTPN11* and *SOS1*, including *LZTR1*, *NRAS*, *SOS2*, *RAF1*, *RIT1*, and *KRAS*, in addition to *SHOC2,* associated with Noonan-like syndrome with loose anagen hair (NS/LAH) and *PTPN11-*gain-of-function mutations associated with NSML (*PTPN11/*NSML); SSRI, Selective Serotonin Reuptake Inhibitor.

**Table 2:** Prevalence of psychiatric disorders in children with NF1 and NSSD compared to the general population.

Psychiatric Diagnosis	NF1 (n/N)	NSSD (n/N)	Children’s Population *	Fisher’s Test (NF1 vs. NSSD)	Binomial Test (NF1 vs. Pop)	Binomial Test (NSSD vs. Pop)
Any Psychiatric Disorder	23/29 (79.3%)	72/94 (76.6%)	13%	*p* = 1.000	*p* < 0.001	*p* < 0.001
ADHD	21/29 (72.4%)	48/94 (51.1%)	11.4%	*p* = 0.544	*p* < 0.001	*p* < 0.001
Anxiety Disorders	11/29 (37.9%)	41/94 (43.6%)	10%	*p* = 1.000	*p* = 0.001	*p* < 0.001
Mood Disorders	1/29 (3.4%)	5/94 (5.3%)	4%	*p* = 1.000	*p* = 1.000	*p* = 1.000
Behavioral Disorders	9/29 (31.0%)	21/94 (22.3%)	7%	*p* = 1.000	*p* = 0.002	*p* < 0.001
Sleep Disorders	7/29 (24.1%)	22/94 (23.4%)	35%	*p* = 1.000	*p* = 1.000	*p* = 0.313

* Report from the National Survey of Children’s Health (NSCH) [15,16]. Abbreviations: *ADHD*, attention deficit/hyperactivity disorder; n, number of participants diagnosed; N, data sample; NF1, Neurofibromatosis type 1; NSSD, Noonan syndrome spectrum disorder; Pop, children’s population.

## Data Availability

Data is unavailable due to privacy restrictions.

## Supplementary Materials

The following supporting information can be downloaded at https://www.mdpi.com/article/10.3390/genes16070843/s1. Additional details regarding participants, recruitment, written consent, and exclusion criteria. Table S1. Psychiatric disorders in children with NF1 and NSSD. Table S2. Influence of cognition on the prevalence of psychiatric disorders in children with NF1 and NSSD.
