# Supplementary material for: Increased Prevalence of Psychiatric Disorders in Children with RASopathies: Comparing NF1, Noonan Syndrome Spectrum Disorder, and the General Population
Source: Genes (Basel). 2025 Jul 19;16(7):843. doi: 10.3390/genes16070843 (PMC12294342; doi:10.3390/genes16070843)
Supplement: Supplementary file 1 [file genes-16-00843-s001.zip › Supplementary Materials.pdf]

Supplementary Materials

Recruitment and written consent

Children with RASopathies were recruited through the Noonan Syndrome Foundation (NS), Children's Tumor Foundation (NF1), and Stanford University School of Medicine's website. Study protocols were approved by the Stanford University School of Medicine Institutional Review Board and followed during all study components. Parents or legal guardians provided informed written consent for their child's participation in the study; participants over the age of 7 submitted an additional written assent. Data for this study was collected from ongoing studies, including remote + in-person visits or remote-only visits, depending on the participant's interest and study requirements.

Exclusion criteria

Exclusion criteria for children with RASopathies in the study included (1) children lacking a diagnosis of NF1 or NSSD (2) premature birth (gestational age <34 weeks), low birth weight (<2000g), and (3) having severe neurological disorders or a sensory impairment impeding study participation. Children with NF1 and NSSD presented clinical genetic information upon participation, confirming the status of their pathogenic variants. Two participants in the NF1 group had a clinical diagnosis, and one participant had an NF1 microdeletion. NSSD pathogenic variants in the study included PTPN11, SOS1, KRAS, LZTR-1, NRAS, RAF1, RIT1, SHOC2 and SOS2 (see Table 1). Participants' gender, race, and ethnicity were ascertained by parents' questionnaires. The sample size did not allow for control for these measures and for different pathogenic variants.

General Children's population data

The National Survey of Children's Health (NSCH) is an annual, nationally representative survey conducted by the U.S. Census Bureau on behalf of the Health Resources and Services Administration's Maternal and Child Health Bureau (HRSA MCHB). It provides comprehensive data on the physical and mental health of children aged 0–17 years, as well as information on health care access, family dynamics, and community environments.

For comparison with the general population in our study, we used data from the 2022–2023 NSCH, which included 109,265 children aged 0–17 years across two waves (54,103 in 2022 and 55,162 in 2023). Mental health questions were administered for children aged 3–17 years, focusing on provider-diagnosed conditions such as ADHD, anxiety, depression, behavioral or conduct problems, and autism spectrum disorder.

Statistical Analysis

FSIQ differences between children with and without each psychiatric diagnosis were assessed using two-sample t-tests. Prior to conducting these tests, we evaluated the normality of FSIQ distributions using the Shapiro–Wilk test, which is a prerequisite for parametric analysis. However, this test is not reliable for groups with fewer than three observations. Therefore, diagnoses with fewer than three cases were excluded from analysis, as normality could not be assessed and t-tests could not be validly applied.
