# Supplementary material for: Increased Prevalence of Psychiatric Disorders in Children with RASopathies: Comparing NF1, Noonan Syndrome Spectrum Disorder, and the General Population
Source: Genes (Basel). 2025 Jul 19;16(7):843. doi: 10.3390/genes16070843 (PMC12294342; doi:10.3390/genes16070843)
Supplement: Supplementary file 1 [file genes-16-00843-s001.zip › supplementary_table.pdf]

Table S1. Prevalence of Psychiatric Disorders in Children with NF1 and NSSD

Psychiatric Diagnosis	NF1 (n=29)	NSSD (n=94)
**Any Psychiatric Disorder**	23 (79.3%)	72 (76.6%)
**Mood Disorders**	1 (3.4%)	5 (5.3%)
**Anxiety Disorders**	11 (37.9%)	41 (43.6%)
**GAD**	3 (10.3%)	9 (9.6%)
**Separation Anxiety Disorder**	5 (17.2%)	19 (20.2%)
**Social Anxiety Disorder**	0 (0.0%)	14 (14.9%)
**Specific Phobia Disorder**	8 (27.6%)	22 (23.4%)
**ADHD**	21 (72.4%)	48 (51.1%)
**OCD**	2 (6.9%)	2 (2.1%)
**Elimination Disorders**	2 (6.9%)	19 (20.2%)
**Sleep Disorders**	7 (24.1%)	22 (23.4%)
**Behavioral Disorders**	9 (31.0%)	21 (22.3%)
**Tic Disorders***	2 (6.9%)	2 (2.1%)
**PTSD***	1 (3.4%)	3 (3.2%)

*11 NF1 participants did not complete the KSADS PTSD evaluation. 1 NS participant did not complete the KSADS Tic Disorders evaluation, and 9 NS participants did not complete the KSADS PTSD evaluation.
Abbreviations: *ADHD*, Attention deficit hyperactivity disorder, *GAD*, Generalized anxiety disorder, *NF1*, Neurofibromatosis type 1, *NSSD*, Noonan syndrome spectrum disorders, *OCD*, Obsessive compulsive disorder, *PTSD*, Post traumatic stress disorder.

Table S2. Influence of Cognition on the Prevalence of Psychiatric Disorders in Children with NF1 and NSSD

Group	Psychiatric Diagnosis	Diagnosis N Yes / No	FSIQ With Diagnosis Mean (SD)	FSIQ Without Diagnosis Mean (SD)	Cohen's d	T-Test Adjusted P-Value
NF1	Any Psychiatric	23 / 6	99.6 (11.9)	95.7 (13.9)	0.32	1.000
	ADHD	21 / 8	100.1 (10.8)	95.4 (15.7)	0.386	1.000
	Any Anxiety	11 / 18	104.8 (8.9)	95.1 (12.7)	0.85	0.182
	GAD	3 / 26	97.3 (4.5)	99 (12.8)	-0.131	1.000
	Separation Anxiety	5 / 24	100.2 (8.4)	98.5 (13)	0.137	1.000
	Social Anxiety	0 / 29	-	98.8 (12.2)	-	-
	Specific Phobia	8 / 21	107.6 (8.6)	95.4 (11.8)	1.103	0.055
	Behavioral	9 / 20	97.9 (11.3)	99.2 (12.8)	-0.106	1.000
	Sleep	7 / 22	104.1 (13.9)	97.1 (11.4)	0.587	1.000
	Elimination	2 / 27	91.5 (14.8)	99.3 (12.1)	-0.64	-
	PTSD	1 / 17	102 (-)	98.2 (13.6)	-	-
	Tic	2 / 27	86 (15.6)	99.7 (11.7)	-1.156	-
	OCD	2 / 27	105.5 (4.9)	98.3 (12.5)	0.587	-
	Mood	1 / 28	93 (-)	99 (12.4)	-	-
NSSD	Any Psychiatric	72 / 21	97 (14.3)	97.4 (10.6)	-0.029	1.000
	ADHD	48 / 45	97.4 (13.6)	96.8 (13.5)	0.047	1.000
	Any Anxiety	41 / 52	97 (15.2)	97.2 (12.2)	-0.011	1.000
	GAD	9 / 84	91.8 (11)	97.7 (13.7)	-0.44	1.000
	Separation Anxiety	19 / 74	95.4 (17)	97.6 (12.5)	-0.163	1.000
	Social Anxiety	14 / 79	98.9 (13.6)	96.8 (13.5)	0.156	1.000
	Specific Phobia	22 / 71	98.2 (13.8)	96.8 (13.5)	0.106	1.000
	Behavioral	21 / 72	100.8 (17.8)	96.1 (11.9)	0.35	1.000
	Sleep	22 / 71	93.7 (15.6)	98.2 (12.7)	-0.336	1.000
	Elimination	19 / 74	94 (16)	97.9 (12.8)	-0.292	1.000
	PTSD	3 / 81	99 (32.5)	96.6 (13.3)	0.17	1.000
	Tic	2 / 90	99 (14.1)	97.2 (13.6)	0.134	-
	OCD	2 / 91	96 (9.9)	97.2 (13.6)	-0.085	-
	Mood	5 / 88	92.8 (14.2)	97.4 (13.5)	-0.338	1.000

"–" There were insufficient numbers in the with- and without-diagnosis groups to conduct the statistical analyses.
*11 NF1 participants did not complete the KSADS PTSD evaluation. 1 NS participant did not complete the KSADS Tic Disorders evaluation, and 9 NS participants did not complete the KSADS PTSD evaluation.
Abbreviations: *ADHD*, Attention deficit hyperactivity disorder, *FSIQ*, Full-Scale Intelligence Quotient, *GAD*, Generalized anxiety disorder, *NF1*, Neurofibromatosis type 1, *NSSD*, Noonan syndrome spectrum disorders, *OCD*, Obsessive compulsive disorder, *PTSD*, Post traumatic stress disorder.
